# Supplementary material for: Causes and consequences of sex-chromosome turnovers in Diptera
Source: Evol Lett. 2026 Mar 12;10(3):253–68. doi: 10.1093/evlett/qrag003 (PMC13231274; doi:10.1093/evlett/qrag003)
Supplement: qrag003_Supplemental_Files [file qrag003_supplemental_files.zip › Supplementary_Datasets.zip!/Supplementary_Datasets/Dataset_2_Graphical_representation_outgroups/Syrphidae_Cheilosia_impressa_chromosomes.pdf]

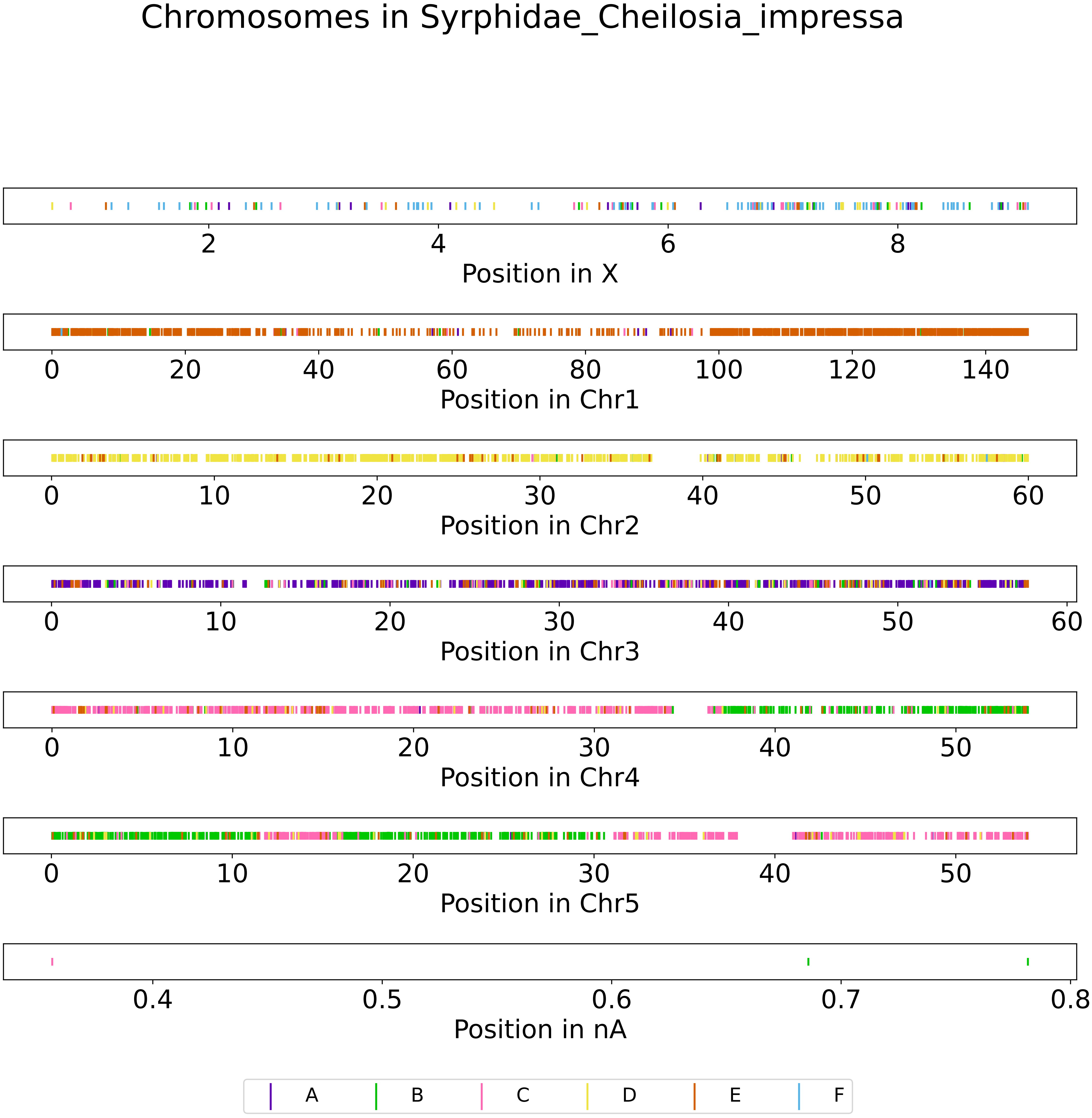
Chromosomes in Syrphidae_Cheilosia_impressa
2
4
6
8
Position in X
0
20
40
60
80
100
120
140
Position in Chr1
0
10
20
30
40
50
60
Position in Chr2
0
10
20
30
40
50
60
Position in Chr3
0
10
20
30
40
50
Position in Chr4
0
10
20
30
40
50
Position in Chr5
0.4
0.5
0.6
0.7
0.8
Position in nA
A
B
C
D
E
F
